# Evaluating the impact of passive driver position on image consistency and repeatability in 3.0T cardiac magnetic resonance elastography

**DOI:** 10.3389/fradi.2026.1912855

**Published:** 2026-10-12

**Authors:** Jun Wang, Shutian An, Zhen Wang, Zichen Hu, Yueyue Zhang, Daiwang Jia, Yong Cheng, Yang Xiang, Ruan Fang, Peiyang Zheng, Zijun Zheng, Zhanglei Li, Yewen Jiang, Yongqiang Yu, Ren Zhao, Xiaohu Li

**Affiliations:** 1 Department of Radiology, The First Affiliated Hospital of Anhui Medical University, Hefei, Anhui, China; 2 Research Center of Clinical Medical Imaging, Hefei, Anhui, China; 3 Anhui Province Clinical Image Quality Control Center, Hefei, Anhui, China; 4 Department of Cardiology, The First Affiliated Hospital of Anhui Medical University, Hefei, Anhui, China

**Keywords:** cardiac magnetic resonance elastography, left ventricular myocardial stiffness, octahedral shear strain signal-to-noise ratio, passive driver, reproducibility

## Abstract

**Background:**

Cardiac magnetic resonance elastography (MRE) is a non-invasive technique that quantifies myocardial stiffness by tracking shear wave propagation through the heart. Shear waves are generated externally and delivered to the thorax through a passive driver placed on the chest. Passive driver position is a critical yet underexplored determinant of measurement quality.

**Purpose:**

To compare two passive driver positions in 3.0 T cardiac MRE — the apical surface projection (ASP) and the left lateral chest wall (LCW) — and to determine which yields more reliable left ventricular (LV) myocardial stiffness measurements.

**Methods:**

Twenty-three healthy volunteers (mean age 43.0 ± 17.0 years; 14 men, 9 women) underwent two cardiac MRE acquisitions, with the passive driver placed first at the ASP and then at the LCW. LV myocardial stiffness and the octahedral shear strain signal-to-noise ratio (OSS-SNR) were measured at both positions. Differences between positions were analyzed using paired t-tests with Cohen's *d*. Inter- and intra-observer reproducibility were assessed using intraclass correlation coefficients (ICC) and Bland–Altman analysis.

**Results:**

Myocardial stiffness was significantly lower at the ASP than at the LCW (9.0 ± 1.7 kPa vs. 14.7 ± 3.0 kPa; mean difference −5.8 kPa, 95% CI −7.2 to −4.3; P<0.001; Cohen's d=−1.73). OSS-SNR was significantly higher at the ASP (1.72 ± 0.48 vs. 1.25 ± 0.29; P<0.001; Cohen's d=0.85). At the ASP, stiffness measurements demonstrated excellent intra- and inter-observer agreement, with ICCs of 0.962 and 0.963, respectively. At the LCW, stiffness measurements demonstrated good inter-observer agreement and excellent intra-observer agreement, with ICCs of 0.839 and 0.914, respectively.

**Conclusion:**

Passive driver placement at the ASP resulted in significantly higher OSS-SNR and superior reproducibility for LV myocardial stiffness measurements compared with LCW placement. Therefore, the ASP should be considered the preferred driver position for cardiac MRE in clinical and research settings.

## Introduction

The mechanical properties of the heart play a central role in cardiovascular health. Increased myocardial stiffness is now recognized as an early and prognostically important feature of several conditions, including myocardial fibrosis, hypertrophic cardiomyopathy, cardiac amyloidosis, restrictive cardiomyopathy, and heart failure with preserved ejection fraction (HFpEF) (1–5). Detecting these changes early can shape treatment decisions and improve long-term outcomes (6, 7). However, most routine imaging tools, including echocardiography, cardiac CT, and standard cardiac MRI, provide only indirect surrogates of stiffness, such as wall thickness, strain, or T1 mapping (8, 9). A method that measures myocardial stiffness directly and quantitatively would close an important diagnostic gap.

Cardiac MRE (magnetic resonance elastography) provides such a technique. A passive driver placed on the chest wall transmits low-frequency mechanical vibrations into the thorax, and phase-contrast MRI captures the resulting shear waves as they propagate through the myocardium. From the wave field, spatially resolved stiffness maps can be reconstructed across the cardiac cycle without contrast administration or invasive pressure measurement (10–12). Cardiac MRE has detected increased myocardial stiffness in cardiac amyloidosis (13), hypertrophic cardiomyopathy, aortic stenosis, and pulmonary hypertension, and its measurements correlate with invasive hemodynamic indices (14). Because of these strengths, cardiac MRE is increasingly regarded as a quantitative imaging biomarker with translational potential.

Reliable cardiac MRE, however, depends on effective shear wave delivery to the myocardium. Unlike the liver or brain, the heart lies behind the ribs and is partly surrounded by air-filled lung tissue, which attenuates and scatters mechanical waves (15–17). In addition, the heart moves rapidly and has a complex three-dimensional geometry. These factors make it challenging to deliver consistent wave energy to the left ventricular (LV) myocardium. In the present single-frequency cardiac MRE protocol, mechanical excitation at one vibration frequency was delivered through a single passive driver. Its position on the chest wall therefore determined where shear waves entered the thorax and how efficiently they reached the LV.

Most published studies place the driver at the apical surface projection (ASP) (15), defined as the region left of the sternum and just above the xiphoid process, based largely on practical convention rather than direct comparison (18). The left chest wall (LCW), at the same axial level as the ASP and along the mid- to anterior-axillary line, represents a clinically feasible alternative site when ASP placement is difficult. In clinical practice, anterior chest wall placement may be limited by factors such as prior thoracic surgery, implanted devices, breast tissue, obesity, or chest wall deformity. The LCW is close to the heart, accessible in the supine position, and compatible with the same fixation band. In contrast, the right chest wall is farther from the heart, and posterior placement is less compatible with routine supine cardiac MRI workflow. Thus, alternative LCW placement represents a practical and recurring clinical consideration rather than a hypothetical concern.

Objective assessment of wave quality is therefore important when comparing passive driver positions. The octahedral shear strain signal-to-noise ratio (OSS-SNR) is a commonly used quantitative metric of shear-wave quality and is associated with the reliability of stiffness estimation in MRE (19). Lower OSS-SNR can reduce the robustness of stiffness inversion and contribute to greater measurement variability. A recent normative study in 125 healthy adults reported that acquisition-related factors, including driver coupling and slice location, are important sources of variability in cardiac MRE (20), and Villalobos Lizardi et al. (21) explicitly identified actuator placement as a key but understudied factor in wave quality. These findings highlight the need to directly evaluate how passive driver location influences myocardial wave quality and stiffness measurement reproducibility.

This study compared two passive driver positions within the same subjects during 3.0 T cardiac MRE: the conventional ASP and an alternative site on the LCW at the same axial level. We evaluated how each position affected LV myocardial stiffness, OSS-SNR, and inter- and intra-observer reproducibility. Our goal was to provide direct within-subject evidence on how passive driver placement shapes cardiac MRE quality and to support evidence-based guidelines for clinical use.

## Materials and methods

### Study design

The study was approved by the institutional review board (PJ-2024-06-90). All participants gave written informed consent. Healthy volunteers were recruited between August 2024 and December 2025. Exclusion criteria included a personal or family history of cardiac disease, MRI contraindications, and pregnancy.

This prospective study tested whether passive driver position affects LV myocardial stiffness measurement in cardiac MRE. Each participant underwent two consecutive scans. The first used the driver at the ASP (left of the sternum, superior to the xiphoid process). Immediately afterward, the driver was moved to the LCW at the same axial level, and the scan was repeated after re-localization.

### Demographics and baseline characteristics

Age, height, weight, and resting blood pressure were recorded for all participants. On the day of scanning, volunteers were asked to avoid strenuous exercise and stimulant-containing food or drink (coffee, tea, chocolate). Each rested supine for at least 30 min before the examination to limit transient blood pressure swings.

### MRE acquisition

Cardiac MRE was employed to quantitatively assess myocardial stiffness in the LV of each subject. An external active driver (Resoundant, Inc., Rochester, MN, US) generated acoustic vibrations, which were transmitted via acoustic tubing to a passive driver in direct contact with the subject.

Each participant underwent two MRE examinations, with the passive driver placed first at the ASP and then at the LCW (Figure 1). For the first scan, the passive driver was coated with acoustic gel and secured at the ASP using an elastic tension band to ensure good coupling between the driver diaphragm and the skin. After approximately 5 min, the passive driver was repositioned at the LCW using the same way, and the MRE scan was repeated. To minimize signal interference from shear wave vibrations, ECG leads for cardiac gating were placed on the posterior aspect of the left shoulder rather than on the chest.

**Figure 1:**
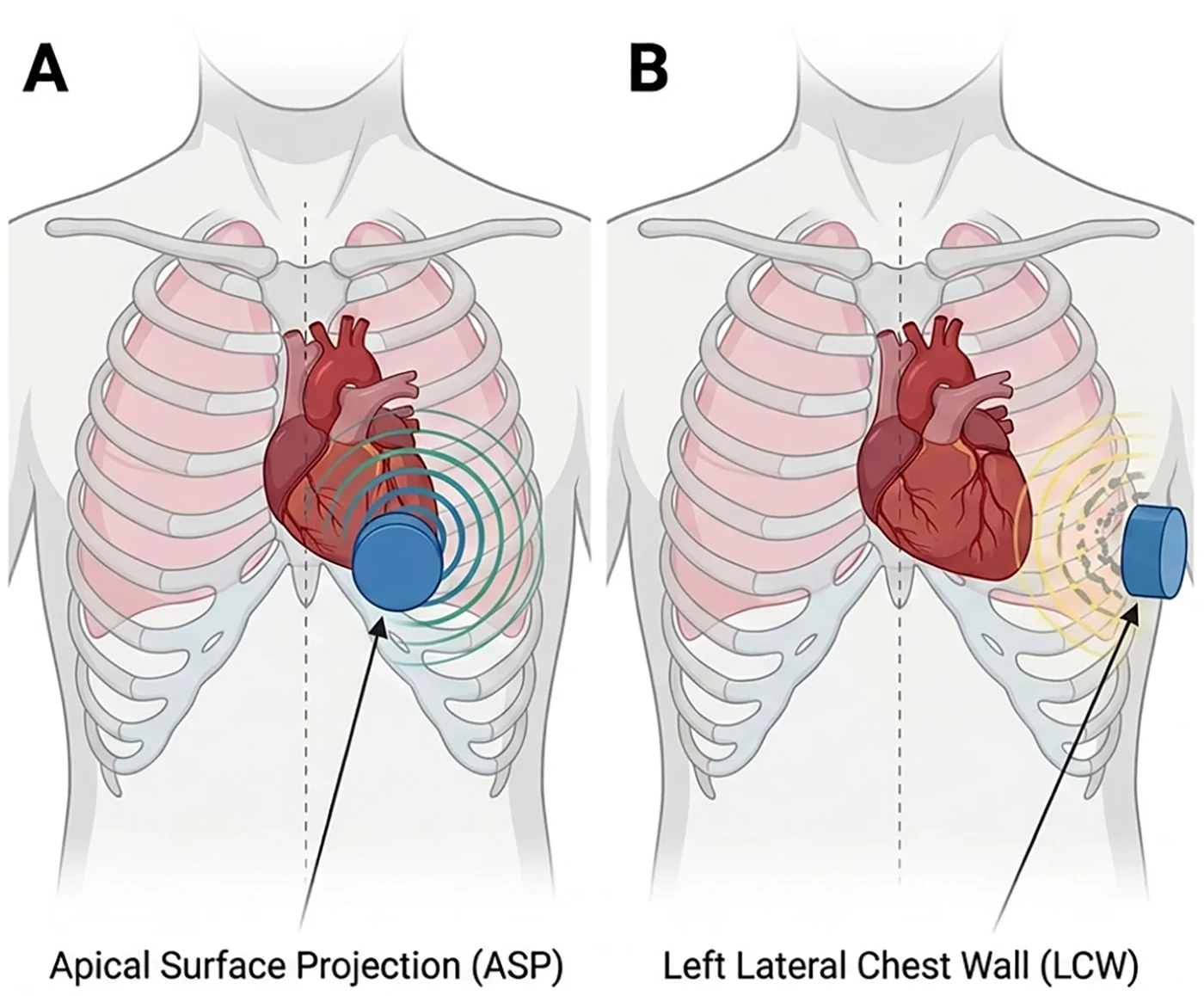
Passive driver placement at the two study positions for cardiac MRE. The passive driver (blue cylinder) was secured at two anatomical locations on the chest wall. **(A)** Driver placed at the apical surface projection (ASP) — left of the sternum, just above the xiphoid process. **(B)** Driver placed at the left lateral chest wall (LCW) at the same axial level. In both configurations, the driver was coupled to the skin with acoustic gel and held in place using an elastic tension band to ensure consistent contact.

Imaging was performed on a 3.0 T closed-bore scanner (Signa Premier 3.0 T, GE HealthCare, Milwaukee, USA), using the AIR body coil array (GEM anterior-posterior array; GE HealthCare). Short-axis MRE images were acquired in the left ventricular short-axis orientation during early systole. A flow-compensated, cardiac-gated, spin-echo, single-shot echo-planar imaging MRE sequence was used with the following parameters: repetition time synchronized to each volunteer's heart rate via ECG gating; echo time 69 ms; field of view 32 cm; image matrix 64 × 64; parallel imaging acceleration factor 2; five contiguous 5-mm-thick short-axis slices; two motion-encoding gradient pairs flanking the refocusing pluse, with a gradient amplitude of 30 m/Tm and matched to the 140-Hz mechanical vibration frequency; motion encoding along the x, y, and z directions; one reference acquisition without motion encoding, denoted as the 0-axis; and four phase offsets evenly distributed over one vibration period. For image processing, the 64 × 64 matrix was reformatted in-house. Images were acquired at the shortest possible delay after the R-wave ECG trigger (approximately 100 ms), the most reproducible phase of the cardiac cycle (21). The effective TR was determined by the cardiac cycle and corresponded to 5 RR intervals. MRE data were acquired during breath-holding to minimize respiratory motion. The acquisition time was approximately 3∼4 min per driver position, resulting in a total MRE acquisition time of approximately 6∼8 min for the two driver positions, excluding the time required for driver repositioning.

### MRE postprocessing and inversion

MRE inversion used the curl of the three-dimensional displacement field together with a three-dimensional local frequency estimation algorithm (22) to invert the wave field and generate shear stiffness maps. This algorithm was chosen for its robustness to noise and its insensitivity to wave reflections. The LV was semiautomatically segmented in commercial software (Cvi42 v5.16.2; Circle Cardiovascular Imaging), and OSS-SNR was calculated from the curl wave fields. The endocardial and epicardial contours of the left ventricular myocardium were manually delineated on the magnitude images. To minimize partial-volume effects from the blood pool and surrounding extracardiac tissues, a fixed 10% inward erosion of the myocardial boundaries toward the mid-myocardium was applied to generate the final myocardial ROI. Regions showing clear boundary-related artifact or nonmyocardial signal contamination on the corresponding magnitude and wave images were excluded. The resulting ROI was transferred to the corresponding elastogram and OSS-SNR map, from which mean myocardial stiffness and OSS-SNR values were extracted (Figure 2). The same ROI criteria were applied to all ASP and LCW datasets. Two readers—a junior radiologist (R1) and a senior radiologist (R2)—independently delineated the LV and OSS-SNR maps. Readers were blinded to each other's measurements and to their prior measurements. R1 repeated the analysis more than 7 days later for intra-observer assessment. Mean stiffness and OSS-SNR were then computed over the remaining volume.

**Figure 2:**
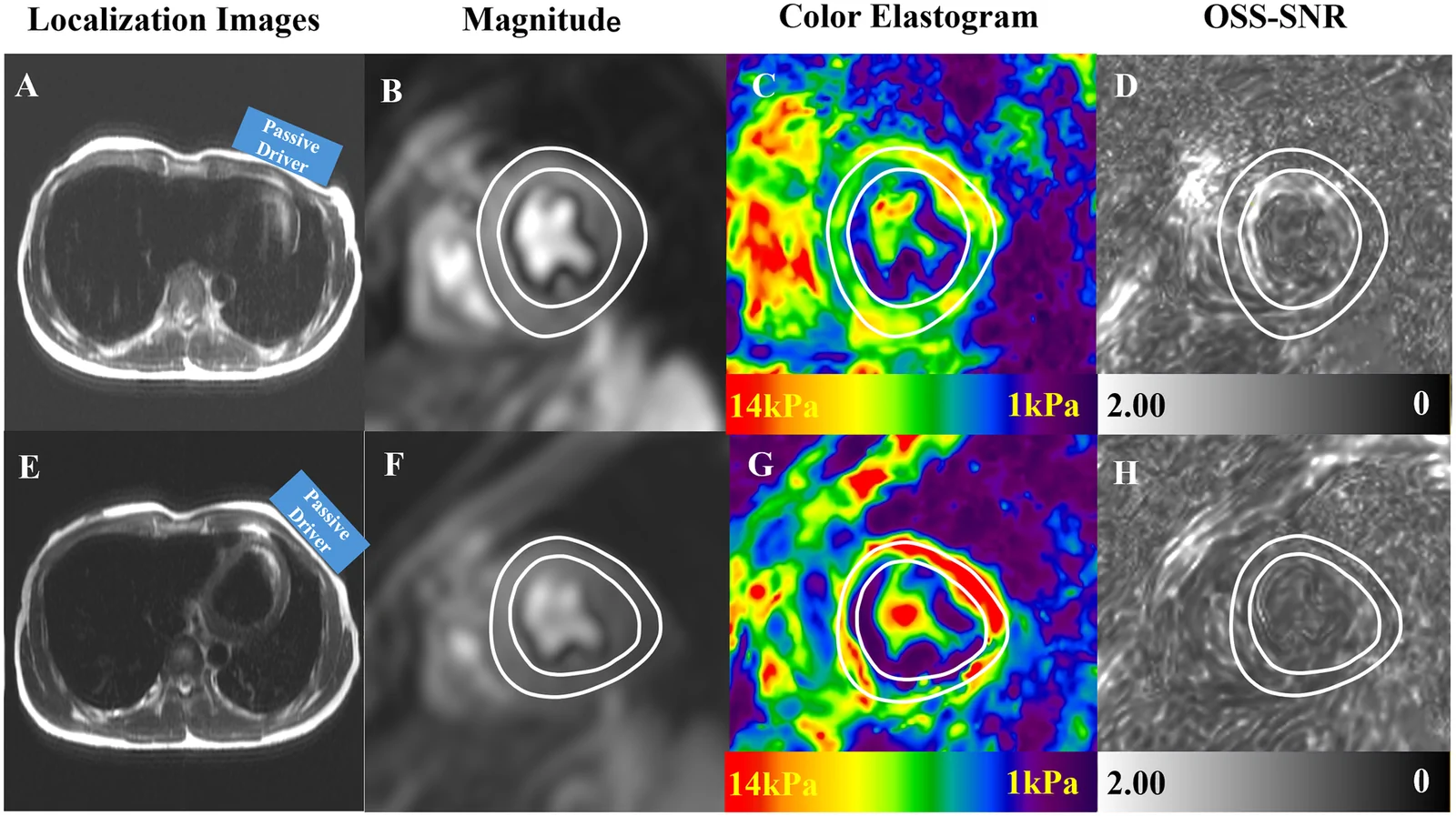
Representative cardiac MRE images from a 24-year-old healthy male volunteer scanned with the passive driver at the ASP and the LCW positions. The top row shows ASP positioning **(A–D)**, and the bottom row shows LCW positioning **(E–H)**. A and E show localization images with the passive driver indicated by the blue rectangle. B and F show MRE magnitude images. C and G show colour-coded myocardial stiffness elastograms using the same colour scale, with stiffness shown in kPa. D and H show OSS-SNR maps using the same scale, with OSS-SNR values shown by the colourbar. In this volunteer, ASP positioning yielded a myocardial stiffness of 7.6 kPa and an OSS-SNR of 1.95, whereas LCW positioning yielded a stiffness of 13.3 kPa and an OSS-SNR of 1.16. ASP, apical surface projection; LCW, left lateral chest wall; MRE, magnetic resonance elastography; OSS-SNR, octahedral shear strain signal-to-noise ratio.

### Statistical analysis

All analyses were performed in R (version 4.4.1; R Foundation for Statistical Computing). Normality was assessed using the Shapiro–Wilk test. Normally distributed variables are reported as mean ± standard deviation (SD); non-normally distributed variables as median (interquartile range, IQR). Stiffness and OSS-SNR values from the two positions were compared using a paired t-test when the differences were normally distributed, or the Wilcoxon signed-rank test otherwise. Effect size was quantified using Cohen's *d*, with values of < 0.2, 0.2–0.5, 0.5–0.8, and > 0.8 interpreted as negligible, small, medium, and large.

Inter-observer agreement was assessed by comparing measurements from the two readers (R1 and R2) in the same session. Intra-observer reproducibility was assessed by comparing R1's two measurements made approximately one month apart. Both analyses used the intraclass correlation coefficient (ICC) with a two-way mixed-effects model, absolute agreement, and single-measures definition. ICC values were interpreted as < 0.50 (poor), 0.50–0.75 (moderate), 0.75–0.90 (good), and > 0.90 (excellent), following Koo and Li (23). The coefficient of variation (CV) was also calculated. Agreement was further examined using Bland–Altman plots, with mean bias and 95% limits of agreement (LoA) reported. A two-sided p<0.05 was considered statistically significant.

## Results

### Participant characteristics

A total of 23 healthy volunteers were enrolled, including 14 men (61%) and 9 women (39%), with a mean age of 43.0 ± 17.0 years and a mean BMI of 23.2 ± 3.2 kg/m^2^. All participants had normal cardiac function on routine cardiac MRI, with a mean LV ejection fraction of 61.4 ± 6.4% and a mean heart rate of 71.1 ± 11.3 bpm. Baseline characteristics of the study cohort are summarized in Table 1.

**Table 1:** Baseline characteristics of the study cohort.

Variable	Overall (*n* = 23)
Sex, *n* (%)	
Male	14 (61%)
Female	9 (39%)
Age (years)	43.0 ± 17.0
Height (cm)	168.3 ± 9.3
Weight (kg)	66.2 ± 13.5
BMI (kg/m^2^)	23.2 ± 3.2
LV EF (%)	61.4 ± 6.4
EDV (ml)	150.4 ± 29.3
ESV (ml)	58.2 ± 15.1
LV Mass (g)	105.4 ± 19.3
EDVI (ml/m^2^)	81.1 ± 11.9
ESVI (ml/m^2^)	31.2 ± 7.2
Heart Rate (bpm)	71.1 ± 11.3
RV EF (%)	53.5 ± 7.9

### Comparison of myocardial stiffness and OSS-SNR between driver positions

Myocardial stiffness and OSS-SNR differed significantly between the two driver positions (Table 2). Mean myocardial stiffness was lower with ASP positioning than with LCW positioning (9.0 ± 1.7 kPa vs. 14.7 ± 3.0 kPa; mean difference, −5.8 kPa; 95% CI, −7.2 to −4.3; p<0.001; Cohen's d=−1.73). In contrast, OSS-SNR was higher with ASP positioning than with LCW positioning (1.72 ± 0.48 vs. 1.25 ± 0.29; mean difference, 0.47; 95% CI, 0.23 to 0.71; p<0.001; Cohen's d=0.85). These findings indicate that ASP placement provided higher wave quality and lower myocardial stiffness estimates than LCW placement (Figures 3, 4).

**Figure 3:**
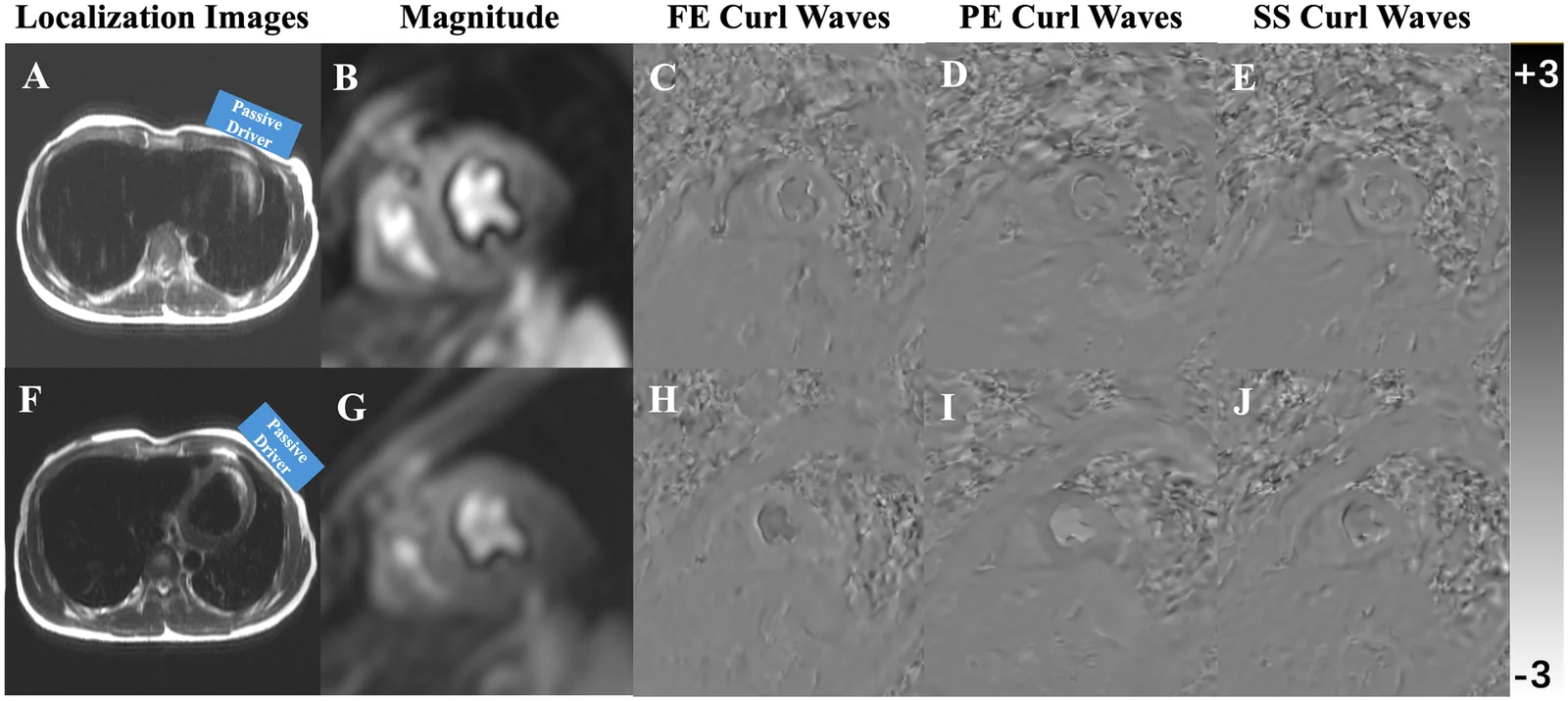
Effect of passive-driver position on the three-dimensional curl wave field in cardiac MR elastography of a healthy volunteer. **(A,F)** Localization images depicting the two passive-driver positions: the apical surface projection (ASP) and the left chest wall position (LCW), respectively. **(B,G)** Corresponding MRE magnitude images acquired at the same imaging location. **(C–E)** and **(H–J)** show the curl-derived shear wave fields obtained from the three orthogonal displacement-encoding directions: frequency-encoding (FE), phase-encoding (PE), and slice-select (SS), for the ASP and LCW driver positions, respectively.

**Figure 4:**
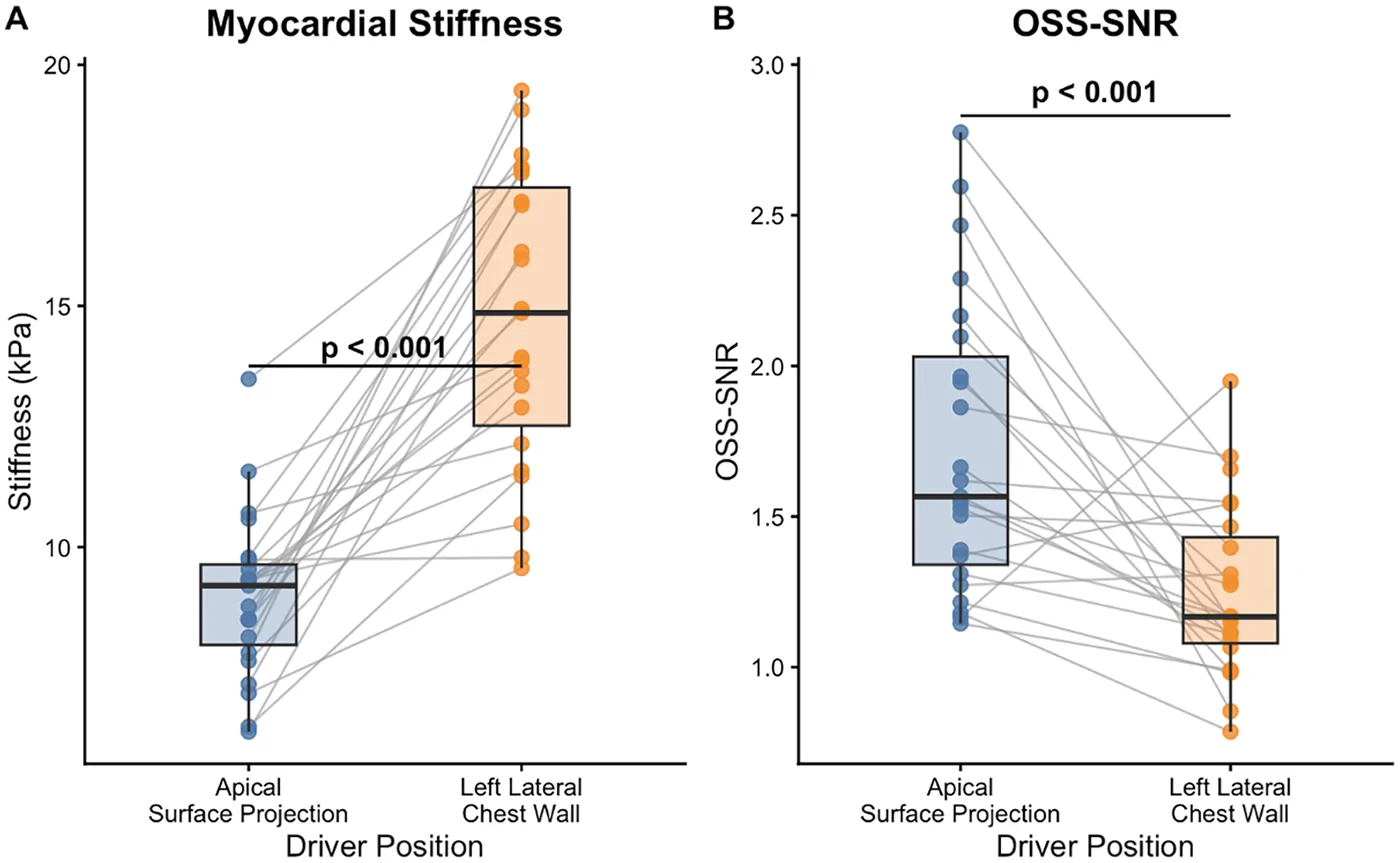
Within-subject comparison of myocardial stiffness and OSS-SNR between the ASP and the LCW. Paired box plots for **(A)** myocardial stiffness (kPa) and **(B)** OSS-SNR at the ASP and the LCW. Each grey line connects measurements from the same participant. Boxes represent the interquartile range, with the horizontal line indicating the median; whiskers extend to the most extreme values within 1.5× IQR. Myocardial stiffness was significantly higher at the LCW than at the ASP (14.7 ± 3.0 vs. 9.0 ± 1.7 kPa; p<0.001), whereas OSS-SNR was significantly higher at the ASP (1.72 ± 0.48 vs. 1.25 ± 0.29; p<0.001).

**Table 2:** Comparison of stiffness and OSS-SNR between driver positions.

Parameter	ASP (Mean ± SD)	LCW (Mean ± SD)	Mean Difference (95% CI)	*P*	Cohen's d
Myocardial Stiffness (kPa)	9.0 ± 1.7	14.7 ± 3.0	−5.8 (−7.2 to −4.3)	< 0.001	−1.73
OSS-SNR	1.72 ± 0.48	1.25 ± 0.29	0.47 (0.23 to 0.71)	< 0.001	0.85

Data are presented as mean ± standard deviation. ASP, Apical Surface Projection; LCW, Left Chest Wall. OSS-SNR, octahedral shear strain signal-to-noise ratio. Mean Difference = Apical−LCW. 95% CI = 95% Confidence Interval. Cohen's d: < 0.2 Negligible, 0.2–0.5 Small, 0.5–0.8 Medium, > 0.8 Large.

### Inter- and intra-observer reproducibility of myocardial stiffness

Reproducibility results for myocardial stiffness are summarized in Table 3, with Bland–Altman plots shown in Figures 5, 6.

**Table 3:** Intra- and inter-observer reproducibility.

Type	Comparison	ICC (95% CI)	CV (%)	*p*-value	Interpretation
Inter-observer	ASP—Stiffness (kPa)	0.963 (0.915–0.984)	4.8%	< 0.001	Excellent
	LCW—Stiffness (kPa)	0.839 (0.659–0.928)	11.4%	< 0.001	Good
Intra-observer	ASP—Stiffness (kPa)	0.962 (0.792–0.988)	3.9%	< 0.001	Excellent
	LCW—Stiffness (kPa)	0.914 (0.725–0.968)	7.5%	< 0.001	Excellent

ASP, Apical Surface Projection; LCW, Left Lateral Chest Wall. ICC, Intraclass Correlation Coefficient (two-way mixed effects, absolute agreement, single measures); CI, 95% Confidence Interval; CV, Coefficient of Variation. Inter-observer: R1 vs. R2 (same session). Intra-observer: R1 1st vs. R1 2nd measurement (1-month interval). Interpretation criteria (Koo & Mae, 2016): < 0.50 Poor; 0.50–0.75 Moderate; 0.75–0.90 Good; > 0.90 Excellent.

**Figure 5:**
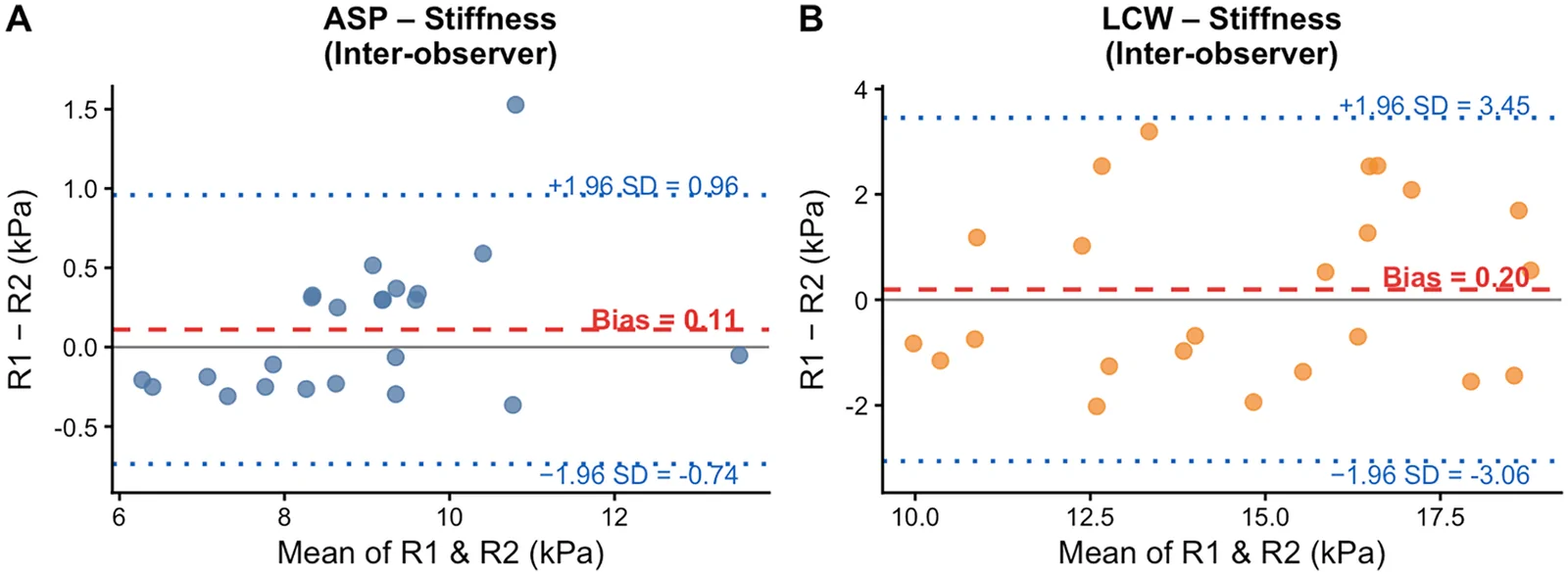
Inter-observer agreement between reader 1 (R1) and reader 2 (R2): bland–altman plots. Plots show measurement differences between R1 and R2 for **(A)** stiffness at the ASP, **(B)** stiffness at the LCW. Inter-observer agreement was excellent for ASP stiffness (bias = 0.11 kPa; 95% LoA −0.74 to 0.96 kPa). Agreement was good for LCW stiffness (bias = 0.20 kPa; 95% LoA −3.06 to 3.45 kPa). The *y*-axis shows the difference between the two readers (R1−R2); the *x*-axis shows their mean. The red dashed line marks the mean bias, and the blue dotted lines mark the 95% limits of agreement (LoA, ±1.96 SD).

**Figure 6:**
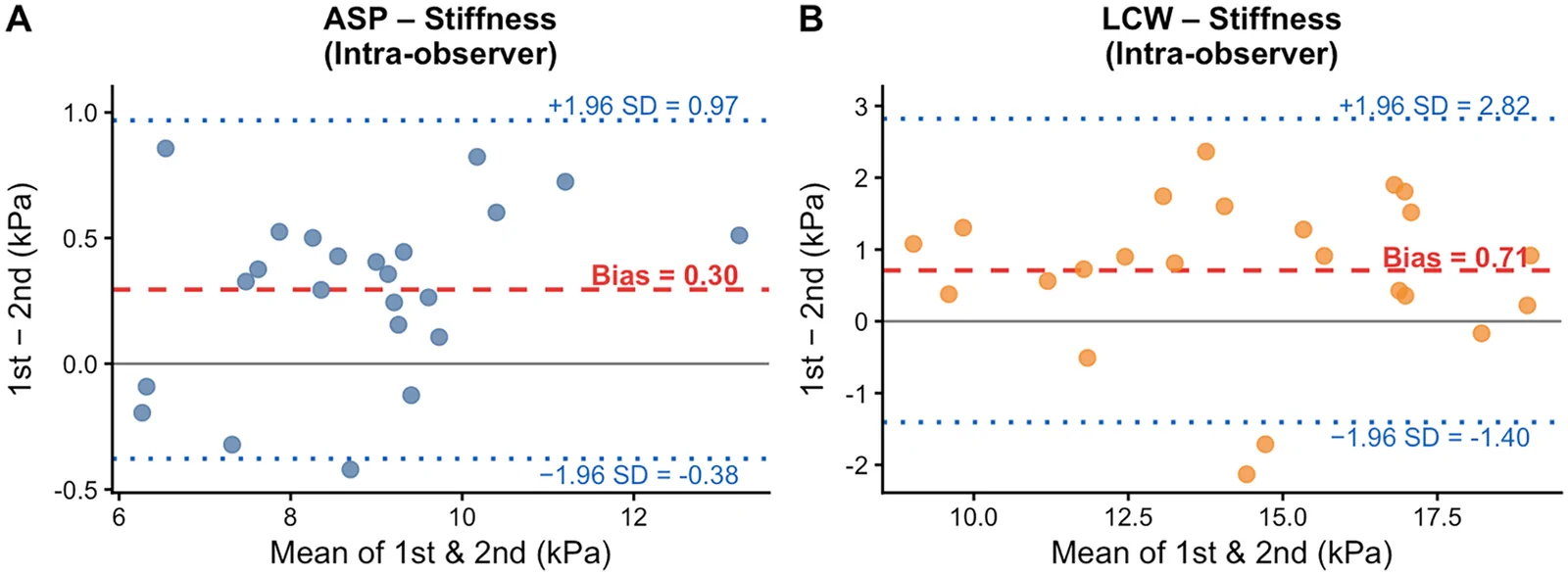
Intra-observer reproducibility for reader 1: bland–altman plots. Plots compare R1's first and second measurements, performed approximately one month apart, for **(A)** stiffness at the ASP, **(B)** stiffness at the LCW. Intra-observer reproducibility was excellent: ASP stiffness (bias = 0.30 kPa; 95% LoA −0.38 to 0.97 kPa), LCW stiffness (bias = 0.71 kPa; 95% LoA −1.40 to 2.82 kPa). The *y*-axis shows the difference between the two measurements; the *x*-axis shows their mean. The red dashed line marks the mean bias, and the blue dotted lines mark the 95% limits of agreement (±1.96 SD).

Inter-observer agreement for stiffness was excellent at the ASP position (ICC = 0.963, 95% CI 0.915–0.984; CV 4.8%; p<0.001). At the LCW position, inter-observer agreement for stiffness was good (ICC = 0.839, 95% CI 0.659–0.928; CV 11.4%; p<0.001). The Bland–Altman plots (Figure 5) show measurement differences between R1 and R2 for stiffness at the ASP and LCW. Inter-observer agreement was excellent for ASP stiffness (bias = 0.11 kPa; 95% LoA −0.74 to 0.96 kPa). Agreement was good for LCW stiffness (bias = 0.20 kPa; 95% LoA −3.06 to 3.45 kPa).

Intra-observer reproducibility for stiffness was excellent at both driver positions: ASP (ICC = 0.962, 95% CI 0.792–0.988; CV 3.9%) and LCW (ICC = 0.914, 95% CI 0.725–0.968; CV 7.5%). Bland–Altman plots (Figure 6) showed small biases for stiffness at both positions: 0.30 kPa at ASP (95% LoA −0.38 to 0.97 kPa) and 0.71 kPa at LCW (95% LoA −1.40 to 2.82 kPa).

## Discussion

This study compared two passive driver locations for cardiac MRE in healthy participants. Placement of the driver at the apical surface projection resulted in lower myocardial stiffness values (9.0 ± 1.7 kPa), more consistent with previously reported normal ranges, along with higher OSS-SNR (1.72 ± 0.48). Both inter- and intra-observer reproducibility were excellent at this location. By contrast, placement at the left lateral chest wall resulted in higher stiffness values (14.7 ± 3.0 kPa) and lower OSS-SNR (1.25 ± 0.29), with good inter-observer agreement for stiffness measurements (ICC = 0.839). These results suggest that the apical surface projection is the more reliable driver location for cardiac MRE in clinical practice.

### Anatomical and mechanical basis for improved ASP performance

Several anatomical and mechanical factors may explain the advantage of ASP positioning. First, the apex is anatomically close to the anterior chest wall and has relatively little intervening lung. Pericardial and epicardial adipose tissue at the apex may also contribute to mechanical coupling, potentially facilitating wave transmission into the myocardium (24). Second, the apex may undergo relatively consistent motion across the cardiac cycle, especially during early diastole when MRE is typically triggered. This relative mechanical stability reduces motion-related artifacts and supports more consistent wave encoding from beat to beat. Cheng et al. (20) recently confirmed that slice location significantly affects myocardial stiffness in cardiac MRE, with apical acquisitions showing lower variability across demographic subgroups. Third, myocardial fiber architecture at the apex may be relatively homogeneous compared with the more complex fiber-orientation changes seen in mid-ventricular and basal segments (25). More homogeneous tissue properties may facilitate wave propagation and support more robust stiffness inversion. Nemavhola (26) reviewed passive and viscoelastic stiffness across measurement scales and noted that regional fiber heterogeneity may influence MRE accuracy. These factors together suggest that the ASP is a mechanically and anatomically favorable site for the passive driver.

### Myocardial stiffness values in the context of prior studies

The mean ASP stiffness of 9.0 ± 1.7 kPa in our healthy cohort is consistent with prior cardiac MRE studies in healthy participants. Cheng et al. (20) reported myocardial stiffness values in healthy adults that varied according to slice location but remained within a comparable range. Arani et al. (3) showed that MRE-derived myocardial stiffness increases with age and differs between sexes, providing a reference framework across diverse populations. Because no independent reference standard for myocardial stiffness was available, the higher LCW-derived stiffness values (14.7 ± 3.0 kPa) cannot be interpreted as proven overestimation. Rather, their coexistence with lower OSS-SNR and wider agreement limits suggests greater susceptibility to wave-quality-related measurement variability. Prior MRE methodological studies have emphasized that reliable stiffness estimation requires adequate wave quality and sufficient strain SNR (2, 27). The octahedral shear strain signal-to-noise ratio (OSS-SNR) has been proposed as a quantitative measure of wave quality in 3D MRE and has been used in cardiac MRE studies to assess the reliability of myocardial wave propagation (19). Low OSS-SNR may reduce the robustness of stiffness inversion and increase measurement variability (12). LCW-derived measurements should therefore be interpreted with caution, particularly when wave quality is reduced.

### Clinical implications of passive driver location

Driver placement is one of the few operator-controlled variables in cardiac MRE, and our study shows that it directly affects image quality and quantitative stiffness measurements. A 5.8 kPa difference between driver locations is unlikely to represent a trivial measurement error and is comparable to, or may exceed, disease-related stiffness differences reported in prior cardiac MRE studies (3, 6, 13, 14). If clinicians rely on myocardial stiffness thresholds to triage patients for biopsy, advanced therapy, or surveillance, inconsistent driver placement could potentially shift a normal examination into an abnormal range. For an imaging biomarker intended to guide management in conditions such as cardiac amyloidosis, and HFpEF (5, 6, 13), this degree of position-dependent bias may compromise clinically interpretability unless acquisition protocols are standardized.

The wider Bland–Altman limits of agreement at the LCW location, particularly for inter-observer stiffness measurements, likely reflect the presence of interposed lung tissue. Air-filled lung parenchyma attenuates shear waves and alters their propagation pathway, producing more heterogeneous wave fields within the myocardium (28). The wave images from our cohort supported this interpretation, showing visibly less uniform shear wave propagation when the driver was placed at the LCW. Irregular wave fields can complicate stiffness inversion and increase reader-dependent variability, consistent with the lower inter-observer ICC observed at the LCW.

The remaining intra-reader and inter-reader variability observed with LCW positioning may reflect the reduced robustness of ROI-based stiffness measurements under poorer wave-quality conditions. Elastograms showed greater spatial heterogeneity at the LCW; therefore, small differences in ROI location could have a larger effect on the mean myocardial stiffness estimate. The parallel reduction in OSS-SNR and increase in stiffness at the LCW suggest that poorer wave quality may bias stiffness estimation upward, rather than merely increasing random measurement variability. This finding further supports the improved robustness of ASP positioning for cardiac MRE stiffness assessment.

The ASP location avoids several of these limitations. The cardiac apex lies directly behind the anterior chest wall with minimal intervening lung tissue, allowing shear waves to travel a shorter path with less attenuation and more homogeneous myocardial coverage. This anatomical advantage likely explains the higher OSS-SNR and better observer reproducibility observed with ASP positioning.

### Reproducibility

Reproducibility is essential for any imaging biomarker entering clinical practice (29, 30), and prior work has demonstrated the scan-rescan reproducibility of 3.0 T single-frequency cardiac MRE for quantifying left ventricular myocardial stiffness (31). Building on this foundation, the present study further highlights that passive driver placement can influence ROI-based measurement variability and, consequently, clinical translatability.

Intra-observer reproducibility was excellent at both driver positions. Inter-observer agreement for myocardial stiffness was excellent at the ASP (ICC = 0.963) and good at the LCW (ICC = 0.839). Although reproducibility remained acceptable at the LCW, the wider Bland–Altman limits of agreement and higher coefficient of variation indicate greater reader-dependent variability under lower wave-quality conditions.

That degree of inter-reader variability may be clinically problematic. Two readers analyzing the same study could reach different diagnostic interpretations depending on subjective ROI choices in a noisy wave field. Castelein et al. (32) similarly emphasized that reproducibility benchmarks, including both interscan and inter-reader reproducibility, must be established before cardiac MRE stiffness can be trusted in routine practice. Our findings extend this concept by showing that standardized ASP placement may reduce reader-dependent variability in ROI-based stiffness measurements. This procedural standardization could improve consistency across centers, scanners, and readers without requiring new hardware or pulse sequences.

Reliable cardiac MRE requires sufficient myocardial wave penetration, relatively homogeneous wave propagation, and adequate wave SNR for robust stiffness inversion. Prior cardiac MRE studies have emphasized these technical requirements for reliable myocardial stiffness assessment. Our study adds to this literature by directly quantifying, within the same subjects, the impact of passive driver location on OSS-SNR, myocardial stiffness estimates, and observer reproducibility. These findings indicate that driver placement should be regarded as a key acquisition parameter rather than a minor procedural detail.

Collectively, these findings emphasize that driver placement should be considered a key component of cardiac MRE protocol standardization, similar to vibration frequency, breath-holding strategy, motion-encoding direction, and inversion algorithm. This may be particularly important for longitudinal monitoring, where position-dependent variability could be misinterpreted as disease progression or treatment response.

### Limitations and future directions

Several limitations should be acknowledged. First, the cohort was limited to healthy volunteers. Whether these findings extend to patients with cardiomyopathy, HFpEF, valvular disease, or amyloidosis remains to be confirmed. In disease states, altered cardiac geometry, increased baseline stiffness, and changes in pericardial anatomy may shift the relationship between driver position and wave delivery. Recent reviews have highlighted that altered myocardial structure and mechanics in hypertrophic cardiomyopathy (HCM) may affect elastographic measurements, which may require position-specific driver adjustments (33). Second, the sample size (*n* = 23) was modest. Larger studies are needed to determine how age, BMI, sex, and chest wall anatomy influence driver-position effect, particularly in patients with obesity, increased anterior chest wall soft tissue, or chest wall deformities. Third, the acquisition order was fixed, with ASP performed before LCW in all participants. Therefore, scan-order effects cannot be fully separated from driver-position effects. Physiological variation, participant fatigue, adaptation to the scanning environment, or repeated positioning may have contributed to the observed differences. Although the findings are consistent with the expected mechanical advantage of ASP positioning, randomized or counterbalanced studies are needed for confirmation. Fourth, only two single-driver positions were compared. The present study was designed as a focused comparison of two practical driver locations that can be readily implemented in a clinical cardiac MRE workflow. However, this design does not determine whether multi-driver, multi-position, multifrequency, or patient-tailored vibration strategies could provide superior wave coverage or more robust stiffness estimation. Meyer et al. (34) demonstrated the feasibility of free-breathing 3D cardiac MRE for spatially resolved myocardial stiffness mapping across the left ventricle. Future studies should determine whether three-dimensional acquisitions, multifrequency excitation, patient-tailored driver configurations or high spatiotemporal resolution can further improve wave coverage and stiffness estimation (35). Finally, all scans used a single vibration frequency (140 Hz) on a single 3.0 T platform, and generalizability to other frequencies, field strengths, and sequence implementations requires separate validation (36, 37).

## Conclusion

Placement of the passive driver at the ASP resulted in more reliable cardiac MRE measurements than placement at the LCW. Stiffness values were closer to established normal reference values, OSS-SNR was higher, and both inter- and intra-observer reproducibility were excellent. Standardizing the ASP as the preferred driver position may provide a simple, low-cost strategy to reduce variability in cardiac MRE across centers and readers. Further validation in patient populations is needed to support clinical translation.

## Data Availability

The original contributions presented in the study are included in the article/Supplementary Material, further inquiries can be directed to the corresponding authors.
